# The Re-Naming of Wards

**Published:** 1923-11

**Authors:** Frank Green

**Affiliations:** St. Bartholomew's Hospital, E.C.1


### The Re-naming of Wards.

SIR,—I see that you refer in your October number to my article "Back to the Gospels," published in the *St. Bartholomew's Hospital Journal* for September, 1923, and that you invite correspondence upon the subject of the renaming of hospital wards to commemorate modern benefactors.

In justice to myself, may I be permitted to open this correspondence by stating here, as I did in that article, that I am as sensible as anybody else of the generosity of the "Men of Smithfield"? Further, that I suggested the putting up of a tablet or memorial in "John" Ward to commemorate their generosity? The thing to which I and many of my colleagues object is the name "Smithfield" which, to our minds, is at once hideous, materialistic, and impersonal. Our objection, therefore, is specific rather than of general application. For that reason, I do not see that any useful purpose will be served by your readers entering upon a correspondence on this subject. That is, however, for them to decide. May I suggest that anybody who seriously contemplates a discussion of my protest should purchase a copy of the *St. Bartholomew's Hospital Journal*, and read the article for himself?

I notice that, in a gallant attempt to change my name into "Smithfield," you refer to me in your paper as "Mr. Smith." Isn't that, perhaps, a little unjust? FRANK GREEN.

St. Bartholomew's Hospital, E.C.1.

